# Parasympathetic autonomic dysfunction is more often evidenced than sympathetic autonomic dysfunction in fluctuating and polymorphic symptoms of "long-COVID" patients

**DOI:** 10.1038/s41598-023-35086-8

**Published:** 2023-05-22

**Authors:** Adrien Zanin, Guy Amah, Sahar Chakroun, Pauline Testard, Alice Faucher, Thi Yen Vy Le, Dorsaf Slama, Valérie Le Baut, Pierre Lozeron, Dominique Salmon, Nathalie Kubis

**Affiliations:** 1 INSERM UMR1144, Université Paris Cité, 75018 Paris, France; 2 Service de Physiologie Clinique – Explorations Fonctionnelles, AP-HP, DMU DREAM, Hôpital Lariboisière, Université Paris Cité, 75010 Paris, France; 3 Department of Immunology and Infectious Diseases, APHP, Cochin-Hôtel-Dieu Hospital, Université Paris Cité, 75004 Paris, France; 4 Service de Physiologie Clinique – Explorations Fonctionnelles, Hôpital Lariboisière, 2 rue Ambroise Paré, 75475 Paris CEDEX10, France

**Keywords:** Cardiology, Neurology, Signs and symptoms

## Abstract

Several disabling symptoms potentially related to dysautonomia have been reported in “long-COVID” patients. Unfortunately, these symptoms are often nonspecific, and autonomic nervous system explorations are rarely performed in these patients. This study aimed to evaluate prospectively a cohort of long-COVID patients presenting severe disabling and non-relapsing symptoms of potential dysautonomia and to identify sensitive tests. Autonomic function was assessed by clinical examination, the Schirmer test; sudomotor evaluation, orthostatic blood pressure (BP) variation, 24-h ambulatory BP monitoring for sympathetic evaluation, and heart rate variation during orthostatism, deep breathing and Valsalva maneuvers for parasympathetic evaluation. Test results were considered abnormal if they reached the lower thresholds defined in publications and in our department. We also compared mean values for autonomic function tests between patients and age-matched controls. Sixteen patients (median age 37 years [31–43 years], 15 women) were included in this study and referred 14.5 months (median) [12.0–16.5 months] after initial infection. Nine had at least one positive SARS-CoV-2 RT-PCR or serology result. Symptoms after SARS-CoV-2 infection were severe, fluctuating and disabling with effort intolerance. Six patients (37.5%) had one or several abnormal test results, affecting the parasympathetic cardiac function in five of them (31%). Mean Valsalva score was significantly lower in patients than in controls. In this cohort of severely disabled long-COVID patients, 37.5% of them had at least one abnormal test result showing a possible contribution of dysautonomia to these nonspecific symptoms. Interestingly, mean values of the Valsalva test were significantly lower in patients than in control subjects, suggesting that normal values thresholds might not be appropriate in this population.

## Introduction

In addition to acute potentially life-threatening symptoms, SARS-CoV-2 infection has been reported to lead to "long-COVID" or "post-acute COVID" in some patients. These patients experience a wide range of symptoms, including unusual fatigue, cognitive and potential autonomic dysfunction. The World Health Organization has defined "long-COVID" as the persistence of at least one initial symptom more than three months after initial infection, not explained by another etiology, with symptoms lasting more than two months^1^.

Some of the symptoms observed could potentially be related to autonomic failure^2–4^. However, extensive autonomic exploration in such patients is rare, and confounding factors, such as concomitant medications, are rarely considered. Postural orthostatic tachycardia syndrome (POTS) is the most frequent form of autonomic dysfunction reported in long-COVID patients^5,6^. POTS is defined by an increase in heart rate (HR) of at least 30 beats per minute (bpm), or to more than 120 bpm within 10 min of standing, combined with orthostatic intolerance and absence of orthostatic hypotension^6^. Other dysfunctions have been reported, such as orthostatic intolerance or hypotension, neurocardiogenic syncope^7^, small-nerve neuropathy^8^, altered pupillary dilation, sudomotor dysfunction, and blunted HR variability^2,3,9^. Fatigue in long-COVID patients has been associated to dysautonomia^10^. These symptoms could result from immune or virus-mediated neuropathy, deconditioning, hypovolemia^3^, post-traumatic stress disorder^11^ or autoimmune autonomic ganglionopathy^7^.

The primary aim of our study, was to perform a systematic evaluation on a population of patients with long-COVID referred for severe disabling long-term manifestations of possible dysautonomia, to identify potential impairment of sympathetic, parasympathetic or of both systems. Moreover, we assessed the sensitivity of these tests routinely used in other etiologies of dysautonomia, and described the characteristics of the patients and their clinical course.

## Methods

### Patients

We prospectively included all patients with long-COVID and severe disabling long-term manifestations, including effort intolerance and possibly related to dysautonomia. They were consecutively referred by the Infectious Diseases and Immunology Department (Cochin Hotel Dieu Hospital) to the Clinical Physiology Department (Lariboisière Hospital) for evaluations of autonomic function at the day hospital. All patients who presented symptoms severely affecting quality of life, with prolonged sick leave (> 1 month, except two), who had not recovered or had not improved by the time of inclusion and accepted the one-day hospitalization for the autonomic evaluation were included from February to October 2020. The study was approved by the local institutional review board of Henri-Mondor Hospital (Ethics Committee Number 00011558, Approval Number 2020-088). Informed consent was obtained from all individual participants included in the study. Exclusion criteria were non-consent and absence of affiliation to a social security scheme.

Initial SARS-CoV-2 infection was considered certain if one of the following criteria was satisfied: a positive reverse transcription-polymerase chain reaction (RT-PCR) test, a positive antigenic test, a positive serological test result, or sudden and sustained anosmia/ageusia or suggestive chest tomodensitometry (CT) findings during the pandemic. SARS-CoV-2 infection was considered probable if a sudden onset of three of the following criteria was observed in the pandemic setting: fever, headache, asthenia, myalgia, dyspnea, cough, chest pain, diarrhea or odynophagia^1,12^.

Controls were age-matched healthy volunteers (colleagues, nursing staff), male and females, who had been infected by the SARS-CoV-2 with a positive PCR or serology for all of them and healed without residual symptoms. They had no past medical history and took no medications. Exclusion criteria were absence of positive SARS-CoV-2 PCR or serology to confirm the infection.

### Demographic and clinical data

Clinical examination was performed by neurologists and one cardiologist involved in dysautonomia investigation. Kale's score^13^, a functional score of autonomic neuropathy, and a composite autonomic scoring scale (CASS)^14^ score were calculated for each patient. The median duration of follow-up was also recorded.

A standardized questionnaire was used to collect the following data at baseline: age, sex, occupation; previous medical history (including allergies, autoimmune disease, chronic disease, asthma, immunosuppression, lifestyle factors and exercise, anxiety and psychiatric history), body mass index, date of infection (date of symptom onset), time between symptom onset and admission, symptoms at acute onset and at the time of evaluation, return to work and usual activities. We also recorded Nijmegen score (hyperventilation syndrome score)^15^, SD12 Scores (The Somatic Symptom Disorder—B Criteria Scale to assess the B criteria of DSM-5 somatic symptom disorder)^16^ and the Hospital Anxiety and Depression Scale^17^. Symptoms at time of the autonomic system evaluation were also collected. At last, the sensation of warm and cold, and of elicited pain, was evaluated to detect potential associated small-nerve fiber neuropathy^18^.

### Evaluation of autonomic function

The adrenergic system (sympathetic) mediates sudomotor function and blood pressure (BP) variation in the standing position and during the 24-h ambulatory monitoring. The vagal system (parasympathetic) mediates HR variability during deep breaths, the Valsalva maneuver and in the standing position. The Sicca syndrome that may be caused by parasympathetic or sympathetic impairment, was assessed with the Schirmer test^19^.

Drugs interfering with the autonomic nervous system, such as beta-blockers and opioids, were withheld and tests performed after five elimination half-lives when possible, and were noted otherwise.

#### Sudomotor evaluation

is mediated by the sympathetic post-ganglionic cholinergic nerves. It was assessed by measuring both the sympathetic skin response (SSR) and electrochemical skin conductance (ESC, Sudoscan®).

The SSR was tested with the same NATUS Dantec Keypoint 2.32 machine used for neurography^20,21^. The test was performed with the patient lying in a quiet warm room. SSR was recorded on both palms and soles after the random application of an electrical stimulus to both wrists. Given the variability of the latency of the response, only absence/presence was considered^20^.

ESC measures the conductance (µS) of the skin resulting from the contact of the palms and soles with stainless steel electrodes. A very low current was applied to the electrodes to induce a change in electrochemical skin conductance based on reverse iontophoresis and chronoamperometry^22^. Conductance was categorized as normal or abnormal according to the measurements.

#### Cardiovascular tests assessing both parasympathetic and sympathetic components of the autonomic nervous system

All values were compared to published thresholds for distinguishing patients from healthy subjects, and to values for age-matched healthy subjects. All tests were repeated three times, and mean values were calculated.

##### Parasympathetic tests

In healthy subjects, HR increases on standing, and decreases on expiration during the deep breathing maneuver and the Valsalva maneuver^23–25^.

On standing, HR increases for the first 15 s, subsequently decreasing to reach its minimal value at 30 s. The normal value for the RR ratio between the first 30 s and the first 15 s is > 1^23,24^.

The HR response to deep breathing was measured during six cycles of inspiration and expiration over one minute, with the patient in a supine position and the number of cycles paced by a metronome or the investigator. In healthy subjects, HR decreases on expiration during the deep breathing maneuver. The RR ratio of HR during the longest expiration to HR during the shortest inspiration was considered normal if > 1.17^24^.

HR response during the Valsalva maneuver was assessed in the absence of contraindications. The patient was asked to exhale into the mouthpiece of a sphygmomanometer at 40 mmHg for 15 s and to breathe normally for one minute thereafter. In healthy subjects, HR decreases during the Valsalva maneuver. The RR ratio between maximal HR at exsufflation and minimal HR after exsufflation is considered normal for values > 1.21^24,25^ and abnormal for values ≤ 1.1.

##### Sympathetic tests

BP and HR were measured after 10 min of quiet rest and 1, 3, 5, and 10 min after standing up. Neurogenic orthostatic hypotension is defined by a decrease in systolic BP (SBP) of 20 mmHg and/or a decrease in diastolic BP (DBP) of 10 mmHg with no significant change in HR (change of less than 15 bpm). In a recent study conducted in neurodegenerative synucleinopathies^26^, a higher sensitivity (91.3%) and specificity (88.4%) than for the classical procedure (79% and 87%, respectively) was reported for a ΔHR/ΔSBP ratio below 0.5 bpm/mmHg, for distinguishing between neurogenic and non-neurogenic orthostatic hypotension. This criterion was preferentially applied.

The patient underwent 24-h ambulatory blood pressure monitoring (24-h ABPM) on the day before admission to the day hospital. BP was recorded every 15 min from 7:00 am to 11:00 pm and every 30 min from 11:00 pm to 7:00 am with an automatic device (Novacor Diasys II Integra), by the Korotkoff method. The parameters evaluated were mean 24-h, daytime and nighttime SBP, DBP, HR, and nighttime SBP reduction. Mean nighttime BP was defined as the mean BP measurement from bedtime until the patient awoke. Mean daytime BP was defined as the mean BP measurement from awakening until bedtime. Nighttime SBP reduction was calculated as the percent decrease in mean SBP during nighttime sleep versus mean SBP during daytime activity: 100 × ([mean daytime SBP − mean nighttime SBP]/mean daytime SBP). Patients were classified as "dippers" (values of between 10 and 20%), "non-dippers” (< 10%), "extreme dippers" (≥ 20%), and "reverse dippers" ([or "risers"], mean nocturnal SBP > mean diurnal SBP). Non-dipping or reverse dipping status is suggestive of dysautonomia^27^. HR was also measured, but only during short intervals during the period of BP recording. The values obtained were therefore considered insufficient for a reliable assessment of the potential lack of HR variability.

#### Schirmer test

The Schirmer test measures the secretory capacity of the lachrymal glands. A test strip that is moistened over less than 10 mm after 5 min constitutes an abnormal result^19^.

#### Kale's score

Kale's score, a functional score for assessing autonomic neuropathy, was used as previously described^13^. It assesses six different types of symptoms: orthostatic intolerance, nausea/vomiting, diarrhea/constipation, urination and erectile dysfunction, and is scored from 0 (severely disabled in each category) to 20 in male subjects and 16 in female subjects in the absence of symptoms.

#### Composite autonomic severity score

The composite autonomic severity score (CASS)^14^ derived from the autonomic reflex screen (ARS)^28^ was used to quantify the severity and distribution of the suspected autonomic failure. Three subdomains are considered in this score: sudomotor (score range: 0–3), cardiovagal failure (0–4) and adrenergic (0–4). The total score ranges from 0 to 10, with values or 7–10 considered to indicate severe autonomic failure^7,14^.

### Nerve conduction studies

**Nerve conduction studies** were systematically performed to explore paresthesia and to detect possible large nerve fiber-associated neuropathy (NATUS Dantec Keypoint 2.32 (2015))^29^. Surface electrodes were used for stimulation and recording.

### Biological tests during autonomic function evaluation and imaging data

Blood tests were performed on admission, for all patients (protein immunoelectrophoresis, fasting glycemia, liver enzymes, TSH, B12 and B9 vitamin levels), together with assessments of transthyretin (TTR) gene mutation, immune antibodies, and viral serology (HIV, HBV, HCV), to exclude possible biases in the interpretation of the results (dysthyroidism, alcoholism, diabetes, amyloidosis in cases of monoclonal gammopathy or TTR mutation). Whole-body CT scans with contrast injection (TDM SIEMENS Somatum Definition Edge, acquisition after the injection of IOMERON 350) and brain 18 F-FDG PET (Camera TEP-CT Siemens Biograph MCT flow after injection of 123 Mbq fluorodeoxyglucose) were performed during initial diagnosis or follow-up. These scans were collected, and additional tests and follow-up were offered to patients when necessary.

### Statistical analysis

Data are expressed as medians and interquartile ranges or means ± standard deviation (SD) for continuous variables. Categorical variables are expressed as absolute numbers and frequencies. The Shapiro–Wilk normality test was used to determine whether data followed a Gaussian distribution. Accordingly, Student's *t* test was performed to compare patients and controls for age and Mann–Whitney *U* tests for heart rate changes on deep breathing, during the Valsalva maneuver and on standing. Effect size was calculated via the Cohen’s *d* (https://www.socscistatistics.com/effectsize/default3.aspx). Fisher’s exact test was used to determine if there was a difference in the prevalence of normal tests between patients with certain SARS-CoV-2 infection and patients with probable infection. Values of *P* values < 0.05 were considered statistically significant.

### Ethical approval

This study was performed in accordance with the dispositions of the 1964 Helsinki declaration and later amendments. It was approved by the local institutional review board of Henri-Mondor Hospital (Ethics Committee Number 00011558, Approval Number 2020-088).

## Results

### Patients and subjects

Ten % of the patients seen at the long-COVID consultation during the period of inclusion were proposed autonomic function evaluation. Four of them did not accept the one-day hospitalization and sixteen patients were finally referred to our department 7–29 months after SARS-CoV-2 infection (median 14.5 months [12.0–16.5 months]). Fifteen of these patients were female (Table 1). The median age of the patients was 37 years [31–43 years]. None of the patients were hospitalized during the acute phase of the infection. Twelve patients (75%) had definite objectively confirmed infection (nine had a positive SARS-CoV-2 RT-PCR/serology test, two had acute isolated agueusia or dysgeusia, and one patient had isolated ground glass opacities in the pandemic setting). The other four (25%) had a probable infection. During follow-up, only two (12.5%) of the 16 patients presented ground glass opacities on chest CT-scan, the abnormality most frequently detected in infected patients^30^. Eleven (68.8%) contracted the infection during the first wave of the pandemic. Initial symptoms are described in Table 1. Moreover, one patient presented anxio-depressive disease (6.3%), two had childhood spasmophilia (12.5%), and another two patients had chronic irritable bowel syndrome (12.5%).

**Table 1:** Clinical characteristics of the cohort of patients and type of symptoms at onset.

**Clinical characteristics**	n [IQR]	%
Female, *n*	15	96.8
Age, years	37 [31–43]	
Occupation		
Health professional, *n*	7(43.8%)	43.8
Other, *n*	9	56.2
BMI, kg/m^2^ (/15)	22.1 [19.8–25.2]	
Allergy	8	50
Respiratory, *n*	5	31.3
Drug, *n*	3	18.8
Other, *n*	2	12.5
Autoimmune disease, *n*	5	31.3
Personal history	3	18.8
Familial history	2	12.5
Tobacco use, *n*	1 (2 former smokers)	
Sport, *n* (/13)	9/13	69.2
**At first SARS-CoV-2 episode**		
SARS-CoV-2 RT-PCR (/14) +	6/14	42.9
SARS COV 2 serology +	7/16	43.8
**Symptoms (n patients)**	**Acute**	
Asthenia	14	87.5
Headache	9	56
Anosmia/dysgeusia	9	56
Dyspnea	10	62
Chest pain and oppression	6	37.5
Cough	13	81
Fever/shivering	11	69
Myalgia/arthralgia	4	25
Odynophagia	7	43.8
Other neurological symptoms	3	18.8
Sensory symptoms	0	0
Ophthalmological symptoms	2	12.5
Other cardiopulmonary symptoms	0	0
Rhinorrhea	8	50
Cognitive symptoms	1	6
Palpitations/tachycardia	5 ara>	31
Auditory symptoms	0	0
Skin/venous symptoms	3	18.8
**Other**		
Nijmegen score elevated > 23/64	14/16	87.5
SSD12 score elevated > 12	7/13	43.8
Brain PET scan hypometabolism	12/15	80.0

Data were available for all 16 patients unless otherwise indicated; n, number of patients; IQR, interquartile range.

The control subjects were 16 age-matched healthy volunteers (colleagues, nursing staff) and 11 were females. All of them presented SARS-CoV-2 infection more than 6 months before the evaluation, confirmed by PCR or serology. None had persistent symptoms after the acute infection. Age did not differ significantly between controls and patients (38.2 ± 7.8 vs. 37 ± 7.2 years old, respectively). One subject was a current smoker.

### Long-term symptoms

At time of evaluation, initial symptoms persisted or new symptoms developed. Fourteen of the 16 patients reported intense sustained and unusual asthenia. Long-term symptoms were diverse and classified as neurological, cardiac/pulmonary symptoms (always associated), digestive and other (Table 2). The most frequent neurological disorders were: concentration deficit disorder ("brain fog") in 12 patients (75%), headache in eight (50%), dizziness in six (37.5%), intermittent tremor in four (25%), myalgia in four (25%), lower and upper limb paresthesia in four (25%), "ataxia" in one (6.3%) and intermittent dysphonia in one patient (6.3%). Medications reported in Table 2 were introduced during the course and follow-up of these patients and therefore after the onset of the disease and the reported symptoms.

**Table 2:** Long-term symptoms.

Patients	Interfering drugs	Neurological	Cardiac and pulmonary	Digestive	Other	Kale’s score	CASS
1	No	Paraesthesia and numbness of hands and feet	Orthostatic intolerance, bradycardia, chest tightness, dyspnea	Constipation	Skin color changes, dysuria	13/16	0
2	No	Concentration and memory disorders, vertigo	Hyperventilation		Pain in all four limbs	15/16	0
3	No	Concentration disorders, tremor on standing, missing words	Tachycardia, palpitations			17/20	0
4	Propranolol, amitriptyline	Myalgia	Palpitations, dyspnea	Epigastric burning sensation	Chills	11/16	1
5	Paroxetine	Myalgia	Bradycardia, malaise			16/16	0
6	Codeine, bisoprolol	Paraesthesia, headache, hypoesthesia, balance disorder, concentration disorders	Chest pain, tachycardia	Alternating diarrhea and constipation	Hypoacousia	15/16	1
7	Midodrine, fluoxetine	Headache, tremor episodes, missing words, abnormal movements, concentration disorders	Orthostatic intolerance	Nausea	Tinnitus	12/16	0
8	No	Concentration disorders, vertigo, ataxia, swallowing difficulties	Chest tightness		Dysuria	15/16	0
9	No	Tremor (effort), weakness, dizziness, paraesthesia, concentration disorders	Orthostatic intolerance, dyspnea	Gastroparesis, constipation	Arthralgia, sicca syndrome, pollakiuria	12/16	0
10	Venlafaxine, nefopam, clobazam	Headache, myalgia, dizziness, concentration and sleeping disorders	Palpitations, chest pain, orthostatic intolerance, dyspnea, cough	Diarrhea	Diffuse pain	10/16	4
11	No	Tremor, headache, concentration disorders, myalgia	Orthostatic intolerance, dyspnea	Epigastric pain, alternating diarrhea and constipation, bloating	Sicca syndrome, urinary leakage	12/16	0
12	No	Concentration disorders, headache, vertigo,	Sinus tachycardia, dyspnea, cough	Epigastric pain, abdominal pain, bloating, alternating diarrhea and constipation	Alopecia, nasal congestion	6/16	1
13	Sotalol, tramadol, hydroxyzine	Dysphonia, paraesthesia, headache, concentration disorders	Tachycardia	Abdominal pain, postprandrial bloating, nausea/vomiting after effort	Itching, eczema	7/16	3
14	Famotidine, ebastine	headache, paraesthesia,	Postural tachycardia, palpitations, dyspnea, postprandrial malaise	Abdominal cramps		12/16	1
15	Duloxetine, amitriptyline, nefopam, famotidine	Headache, concentration disorders, irritability, hypersomnia (17 h/day)	Chest pain, dyspnea (at effort)	Diarrhea, postprandrial abdominal pain, anorexia, constipation	Blurred vision, alopecia, urticaria	10/16	0
16	No	Vertigo, insomnia, abnormal movements of left hemibody	Positional and postprandrial malaise, tachycardia, sensation of chest tightness	Vomiting, abdominal pain, dyspeptic syndrome with early satiety	Hypersudation, sicca syndrome, frostbite	12/16	0

Kale’s score ranges from 0 (severely disabled) to 20 (male) or 16 (female) in normal subjects. CASS, Composite Autonomic Severity Score.

All patients reported effort intolerance. Dyspnea was the most prevalent cardiopulmonary symptom, being detected in eight patients (50%). Tachycardia was found in six patients (37.5%), orthostatic intolerance was observed in five patients (31.2%), and palpitations occurred in four patients (25%). The digestive symptoms reported were abdominal pain in six patients (37.5%), constipation in six patients (37.5%), and diarrhea in five patients (31.2%). The symptoms were postprandial in four patients (25%): two had postprandial malaise, one had postprandial bloating and the remaining patient had postprandial abdominal pain.

Other symptoms clinically suggestive of dysautonomia were skin and eye dryness, urine and stool sphincter dysfunction, hot flashes, a dysregulation of sudation dysregulation and shivering. Interestingly, most patients reported an exacerbation of their symptoms after effort.

The Hospital Anxiety Depression Scale was performed in 12 patients out of 16 and six of them had abnormal anxiety scores (Table 3).

**Table 3:** HAD (Hospital, Anxiety Depression scale), A, is for the anxiety score and D, for the depression score.

Patients	A score	D score	Global score
1	4	3	7
2	ND	ND	ND
3	8	1	9
4	9	9	18
5	15	14	29
6	5	3	8
7	ND	ND	ND
8	13	4	17
9	ND	ND	ND
10	14	14	28
11	4	7	11
12	ND	ND	ND
13	10	7	17
14	6	2	8
15	7	14	21
16	12	6	18

Values ≤ 7 indicate normal scores, and values ≥ 11 indicate probable anxiety and depression disorders for each separate score. For the global score, Values ≤ 14 indicate normal scores and values ≥ 15 indicate probable anxiety and depression disorders. *ND* not done.

### Clinical examination

Neurological clinical examination was normal for all but one patient. The exception was patient 15 who reported non-systematized hypoesthesia of the whole body. One patient (6.3%) had ataxia with functional characteristics^31^. Four patients had a tremor (25%). Two of these patients underwent tremor recording in our department, which revealed a functional tremor, and the other two reported tremor after effort, which was confirmed by the physician's observations. Cardiac examination was abnormal in six (37.5%): one patient had isolated orthostatic hypotension with concomitant tachycardia, potentially accounting for orthostatic malaises (patient 1), one patient was diagnosed with orthostatic tachycardia not fulfilling the criteria for POTS (patient 11), one patient (6.3%) was diagnosed with POTS (patient 14) and three presented isolated sinus tachycardia (Tables 2, 3).

Kale's score ranged from 6 to 17 (median = 12 [10.75–15]). Six patients (37.5%) were on medication that might have interfered with orthostatic blood pressure and heart rate measurements and that could not be stopped before testing (Table 2). However, none of these six patients had symptoms in relation with this medication. Six other patients, without interfering medication, presented orthostatic hypotension, sinus tachycardia, orthostatic tachycardia or POTS.

Schirmer test results were abnormal for three patients (18.8%), but who used antidepressant or analgesic drugs in all cases (Table 4), and therefore could not be interpreted as a biomarker of dysautonomia per se.

**Table 4:** Detailed results for autonomic testing, Sudoscan and cardiological expertise (HR, heart rate; NP, not performed, patient unable to perform the maneuver correctly); POTS, postural orthostatic tachycardia syndrome; N, normal; AN, abnormal).

Patients	Certain (C) or probable (P) SARS-CoV-2 infection	Deep breath test	Valsalva	Heart-rate changes on standing	Orthostatic hypotension	Sudoscan/SSR	Cardiologist expertise	Schirmer (mm) (left/right)
1	C	N	N	N	Presence with increased heart rate	N/N	Orthostatic hypotension without dysautonomia	18/20
2	P	N	NP	N	N	N/N	N	19/18
3	C	N	N	NP	N	N/N	N	> 15/15
4	P	AN	N	N	N	N/N	Sinus tachycardia	1/1
5	C	N	N	N	N	N/N	N	> 15/15
6	C	N	NP	N	N	AN (2 hands) N/N	N	> 15/15
7	P	N	N	N	N	N/N	N	20/20
8	C	N	N	N	N	N/N	N	14/25
9	C	N	N	N	N	N/N	N	15/23
10	C	AN	N	N	N	AN (4 limbs)/AN (4 limbs)	Sinus tachycardia	20/20
11	C	N	N	N	N	N/N	Orthostatic tachycardia (no POTS)	20/20
12	C	N	N	AN	N	N/N	Sinus tachycardia	20/19
13	C	AN	AN	N	N	Nl/AN (2 feet)	N	0/0
14	C	AN	N	N	N	N/N	POTS	35/20
15	C	N	N	N	N	N/N	N	2/1
16	C	N	NP	N	N	N/N	N	15/25

Deep breath test, Valsalva and heart-rate changes on standing explored the parasympathetic nervous system; orthostatic hypotension, Sudoscan and SSR explored the sympathetic nervous system. Schirmer test is normal when the test strip is moistened over 10 mm after 5 min.

### Biological and imaging data

During evaluations at the day hospital, all biological test results were normal, with no TTR gene mutations detected in any of the patients. Thoraco-abdominal CT-scans were performed on all patients except patient 3. Two of the CT-scans (12.5%) revealed pulmonary ground glass opacities, basal atelectasis was found in one patient and the images were normal for the other 13 patients. Nerve conduction study (NCS) results were normal for all patients, including the patient who reported four limbs paresthesia.

Brain 18F-FDG PET-TDM had been performed in 15 patients (93.8%) before admission. The results were normal for two patients, one patient presented hypermetabolism in the brainstem and olfactive bulbs, and the other 12 patients displayed hypometabolism, in the brainstem (pons) for 9 patients (56%), the cerebellum for five patients (31.3%), the olfactive bulbs for one patient (6.3%) and the hippocampi or temporal lobe for seven patients (37.5%).

### Autonomic testing

#### Parasympathetic tests

Parasympathetic cardiovascular test results were abnormal for five of our 16 patients (31.2%). HR variation was altered in the deep-breathing test for four patients, the Valsalva test for one patient and the orthostatic test for one patient. Only one patient had altered results in two parasympathetic tests (patient 13). It was not possible to perform the Valsalva maneuver correctly in three patients (18.8%). No significant differences in HR variations were observed for the deep-breathing maneuver or on standing. Interestingly, the score for the Valsalva maneuver was significantly lower in patients (1.4 ± 0.3, *n* = 13) than in controls (1.8 ± 0.6, *n* = 16) (*p* = 0.013) (Cohen's *d* = 0.85, which indicates a high effect size), whereas the mean values obtained in the other two tests did not differ significantly from those for the controls (1.3 ± 0.2 vs. 1.3 ± 0.1 for deep breathing respectively, Cohen’s *d* = 0.17; and 1.2 ± 1.1 vs. 1.12 ± 0.2 for the orthostatic test respectively, Cohen’s *d* = 0.54). The fact that the mean value of the Valsalva score was lower in the long-COVID population than in the healthy control subjects, whereas only one patient had a score below the threshold value that cannot reasonably account for this statistically significant difference between means, suggests that the overall Valsalva score was globally lower in the long-COVID population but without reaching the abnormal threshold for each individual value.

#### Sympathetic tests

None of the patients had neurogenic orthostatic hypotension. Three patients (18.8%) had an altered sudomotor response, whereas none of the controls displayed such changes. The sympathetic skin response was altered in patients 10 and 13 and SudoScan® results were abnormal for patients 6 and 10. We obtained 24-h ABPM recordings for 13 of the 16 patients. Only two patients (patients 13 and 16) had non-dipping status, but their quality of sleep was poor, so interpretation was not unequivocal.

The CASS score of these six patients ranged from 1 to 4 (mild autonomic failure for five, and moderate autonomic criteria for one patient, with no patient satisfying the criteria for severe dysautonomia) (Table 2).

*In summary*, six (37.5%) patients presented either an alteration of the sudomotor response (sympathetic evaluation) or abnormal results for one parasympathetic cardiovascular autonomic test (Table 4). Both types of abnormality were present in two patients (12.5%). In addition, two of them had dry eyes at the Schirmer test. Among these patients, three of them presented sinus tachycardia, and one POTS. There were no alteration of the cardiac autonomic functions in the last two patients who had an orthostatic hypotension but who did not fulfill the POST criteria or neurogenic hypotension criteria (patients 1 and 11). Therefore these two patients were not considered to have a cardiac dysautonomia according to our criteria. The prevalence of normal tests results was not significantly different between patients with certain SARS-CoV-2 infection and patients with probable SARS-CoV-2 infection.

Concerning the 16 control subjects, three had an abnormal Schirmer test, four had abnormal hands responses at Sudoscan (but normal feet responses), two had results slightly below the lower threshold for the heart rate modulation at deep breathing (1.14 and 1.16 for a normal ≥ 1.17) and three had abnormal results for the heart rate modulation at standing (0.95, 0.96 and 0.83). Valsalva was normal for all of them. None had orthostatic hypotension, bradycardia or tachycardia.

### Course of the illness

All patients had fluctuating symptoms, with remissions and relapses; 226 days [54.5–377.8] after the autonomic function evaluation, nine patients (56.25%) had improved (including three on ivabradine), five (31.3%) were stable (including one on ivabradine), and two (12.5%) displayed a worsening of autonomic function (including one on ivabradine). Ivabradine was specifically introduced *after* the one-day admission to the Clinical Physiology Department in five patients: for orthostatic hypotension without dysautonomia in one, sinus tachycardia in three, and POTS in one. One patient was switched onto this drug from propranolol. All patients were encouraged to do isometric exercise and to drink enough water to ensure that they were well hydrated. Five patients were treated with antidepressants and/or anxiolytics during follow-up (Table 2), and three had a normal evaluation for all tests except one who had isolated dry eyes but not dry mouth.

## Discussion

We systematically investigated potential autonomic dysfunction in long-COVID patients suffering severe impairments due to long-lasting and fluctuating disabling symptoms in a prospective single-center study with consecutive patient inclusion. We found that 37.5% of the patients had at least one abnormal test result, associated with mild autonomic failure in 83% and moderate autonomic failure in 16%, with the cardiovascular system and sudomotor function principally affected. These rates are about half of those reported for a previous study based on similar investigations. Shouman et al.^7^ reported that 63% of their 27 patients displayed failures of autonomic function following COVID-19 infection. Interestingly, this autonomic failure affected the various components or the global evaluation in similar proportions to those for our population. Shouman et al.^7^ reported alterations of cardiovagal function in 27% of patients and in the cardio-adrenergic test in 7% of patients, versus 31% and 6%, respectively, in our population. The quantitative sudomotor axon reflex was affected in 36% of their patients while sudomotor function was altered in 18.7% of ours. They began autonomic testing much sooner after the onset of COVID-19 symptoms (4 months versus 14.5 months in our study). No mention was made of drugs potentially interfering with autonomic testing in this previous study and the autonomic testing was not described in detail.

All the tests used here are validated for and used in diabetes mellitus and neurodegenerative diseases, but their sensitivity and specificity may vary with etiology. Consensus recommendations have recently been published^32^. A battery of autonomic tests, including cardiovascular adrenergic, cardiovagal, and sudomotor function, should be performed, with the results interpreted by an expert capable of identifying multiple biases. A final diagnosis of autonomic dysfunction should result from a congruent assessment of the results of autonomic function testing, clinical examination and medical history, because the various symptoms of dysautonomia are not specific and may be encountered in many organic and non-organic diseases. For instance, dry eyes assessed at the Schirmer test, are very common in healthy subjects as shown in our controls, the most frequent cause being a poor blinking habit while reading or looking at a computer screen for long periods of time. Concomitant medication should be reported, and might account for the sicca syndrome reported in this cohort but not for all the other abnormal tests. Among the five patients under antidepressant therapy, only two had abnormal autonomic function test results that may question the imputability of the medication in these results.

The number of pathological tests required remains a matter of debate, with specific assessment principally for diabetes and synucleopathies. For example, one pathological test is sufficient for the diagnosis of possible cardiac autonomic neuropathy in diabetes^33^, but the confirmation of dysautonomia, including at least neurogenic hypotension, requires at least two pathological tests in one domain or one test giving abnormal results in at least two different domains^33–36^. Other authors have suggested that two of the four tests usually performed to investigate the cardiac autonomic system should give abnormal results for a diagnosis of dysautonomia to be established^36^. Using this last definition, five (31%) of our patients had at least two different abnormal tests results. Moreover, criteria may differ between centers^37^. Given the potential confounding factors for these measurements, including room conditions and equipment settings, each laboratory should have its own reference values measured in the same conditions, in accordance with recommendations for good laboratory practice. In that study, we used this battery of autonomic tests including cardiovascular adrenergic, cardiovagal, and sudomotor function.

Sympathetic sudomotor cholinergic evaluation is recommended, preferably with two complementary tests: the quantitative sudomotor axon reflex (QSART), which evaluates the functional integrity of the postganglionic sympathetic sudomotor axon and has been shown to be both reproducible and sensitive in diabetic patients^38^, and the thermoregulatory sweat test, which has the advantage of detecting central and peripheral abnormalities along the sudomotor axis. The combination of these two tests makes it possible to identify the site of dysfunction. The main disadvantage of this approach is the sensation of heat provoked and the limited ability of patients to endure the test. The sympathetic skin response and the ESC tests are much better tolerated, but were found to have a much poorer sensitivity, at 33.3% (specificity of 77.6%) and 49.4% (specificity of 92.5%), respectively for various diseases, including familial amyloid neuropathy with TTR mutation, monoclonal gammopathy, primary Sjögren's syndrome, Fabry's disease and autonomic dysfunction of unknown cause^20^. ESC performs better in diabetes mellitus, with a sensitivity of 76–87.5% and a specificity of 85–76.5%^39,40^. Receiver operating characteristics curves for foot ESC and skin biopsy with an abnormal Utah Early Neuropathy score as the gold standard gave similar areas under the curve^39^. This indicates that despite the relatively high sensitivity of tests performed in diabetes, sensitivity is lower for other diseases potentially involving the ANS. Abnormal responses were recorded for only 18.75% of our patients. Despite the use of a combination of SRR and Sudoscan methods, we cannot rule out the possibility that we have underestimated the true proportion of patients with abnormalities. Papadopoulou et al.^41^ did not use a binary interpretation like that used here (presence or absence). Instead, they compared 11 long-COVID patients with age-matched healthy controls, revealing significantly higher SSR latencies in patients.

We detected no neurogenic hypotension in this cohort of patients. Orthostatic intolerance (not satisfying the criteria for neurogenic orthostatic hypotension or POTS), sinus tachycardia and POTS were the most prevalent syndromes in this cohort of patients with long-COVID, as in other previous studies^3,8^. It has been suggested that POTS^42^ is related to autonomic failure^6^, given that 50% of the patients diagnosed with POTS had a suspected associated small-fiber neuropathy on the quantitative sudomotor axon reflex test (QSART)^43^, but not on skin biopsy. Only one true case of POTS (6.3%) was identified among our patients, but the prevalence of this condition is highly variable, ranging from 0 in 180 patients^44^ to 75% in 20 patients with long-COVID^4^. Norcliffe-Kaufmann et al.^27^ suggested that non-dipping or reverse dipping status is suggestive of dysautonomia, possibly due to the absence of a decrease in sympathetic noradrenergic outflows and the lack of an increase in cardiovagal outflow overnight^45^. We therefore performed 24-h ABPM in 13 of the patients of our cohort but all the results obtained were normal. Isolated sinus tachycardia is difficult to interpret when isolated because it might be due to a white coat effect. In our cohort, we considered this result as it was always associated with an abnormal autonomic function test result.

Parasympathetic tests more frequently gave abnormal results. Sensitivity and specificity of the Valsalva and HR variability to deep breath maneuvers both have a good sensitivity and specificity (≥ 80% for each)^46–48^. This may explain why abnormal parasympathetic cardiac assessment results are more frequent than abnormal sympathetic cardiac assessment results. In our cohort, 37.5% patients had an abnormal value in parasympathetic tests. Moreover, mean values for the Valsalva maneuver differed significantly between patients and controls. Papadopoulou et al.^41^ showed that the cross-sectional area of the vagus nerve was enlarged on ultrasound scans; this finding requires confirmation in larger positive control populations but this approach may constitute an interesting alternative to investigate these patients. In this context, Asarcikli et al.^49^ demonstrated parasympathetic overtone and greater HR variability on 24-h ambulatory electrocardiography (ECG) recordings obtained > 12 weeks after the diagnosis of COVID-19 in patients relative to healthy age- and sex-matched controls. We then hypothesized that the test sensitivity could differ between long-COVID patients and other diseases classically associated with dysautonomia (e.g. diabetes and neurodegenerative diseases) and therefore that the threshold for abnormal values might not be appropriate. To answer this question, we compared mean values between long-COVID patients and healthy subjects. We found a significant lower mean value in the Valsalva test in long-COVID patients, despite the fact that only one of them had abnormal values as defined by a value below ≤ 1.1. This finding will have to be confirmed in a validation study all the more three patients of our cohort were unable to perform the test.

In our study, five out of 16 patients had one or a combination of abnormal parasympathetic tests. Considering that we found no neurogenic hypotension, which is very common in case of diabetic dysautonomia or in neurodegenerative disorders, and that only one patient had both Sudoscan and SSR alterations and only two patients, one or the other positive tests, our results suggest that the parasympathetic tests were most often abnormal in that population, as shown by others^7^. We cannot rule out that tachycardia might be the sole expression of an associated sympathetic dysfunction that we cannot assure. Notably, the three patients with sinus tachycardia and the patient with the POTS syndrome all had in addition an abnormal parasympathetic test.

More generally, we cannot rule out that anxiety may account for some of the symptoms. Among the six patients who had abnormal values at the anxiety score, half of them had a normal autonomic function testing. If anxiety cannot explain all of our results, it might have interfered in some patients with the autonomic function testing.

Barizien et al.^10^ used the nociception level (NOL) index, a composite index that simultaneously integrates HR, photo- plethysmography and skin conductance parameters used to estimate nociception levels in anesthetized patients. It reflects HR variability and parasympathetic/sympathetic balance. They found a significant difference between long-COVID patients with fatigue and long-COVID patients without fatigue, but additional studies are needed to assess whether this tool could be suitable for use at individual level.

Brain 18F-FDG PET-TDM was performed in 15 of the 16 patients and the results were abnormal in 13 patients (87%). Pontic hypometabolism was the predominant feature (60%). Other sites of hypometabolism, such as the hippocampal and olfactive gyri, were also identified, as previously described in long-COVID^50,51^. The relationship between this hypometabolism, symptoms and COVID-19 remains unclear. In Parkinson's disease (PD) 18-PET TDM is considered in differential diagnosis between PD and atypical forms of degenerative parkinsonism (e.g. multiple system atrophy or MSA), with interpretation always in relation to clinical findings^52^. In PD patients with associated dysautonomia, hypometabolism affected completely different regions, such as the parieto-occipital areas, and hypermetabolism, relative to patients with Parkinson's disease without dysautonomia, was detected in the pallido-thalamic, pontocerebellar, inferior frontal, and primary motor areas^53^. Similarly, in MSA with predominant parkinsonism, hypometabolism relative to healthy controls has been detected in the bilateral frontal cortices and striatum^54^. Interestingly, a decrease in regional uptake similar to that observed in long-COVID syndrome has been reported in patients with post-traumatic stress disorder (PTSD)^55^, which partly involves the limbic system^56^. Whether these features can be characteristic of central autonomic dysfunction is still a matter of debate.

Our study has several limitations. As our aim was to focus on examinations that are easy to perform, non-invasive and well tolerated, other invasive tests such as ^123^I-meta-iodobenzylguanidine (MIBG) cardiac scintigraphy^57^, orthostatic catecholamine determination^58^ and laser-evoked potentials^59^ were not performed, and we cannot rule out the possibility that such tests might have been more efficient. Moreover, the patients followed here were seen late in the course of the disease. A larger number of tests could have been positive if they had been performed earlier, however, these patients still reported severe disabling symptoms at the time of evaluation. Another limitation is the "COVID status" of these patients. Not all the patients infected during the first wave of the pandemic had RT-PCR-confirmed diagnoses, due to the lack of available tests. Two of the patients without a positive RT-PCR result had positive serological results when this could be performed during the course of the disease, and one patient had suggestive ground glass opacities on chest CT-scan. These patients still met the criteria for probable COVID-19, as defined in the “Methods” section, but we cannot rule out the possibility of these patients being infected with another virus. Our findings also suggest that usual thresholds for these tests are maybe not appropriate for this population of "long-COVID" patients. Finally, this cohort was of modest size, and four patients among the patients who met the inclusion criteria did not accept the one-day hospitalization, which may constitute a bias. However, all patients were investigated in the same way and the results obtained were compared not only with published normal values, but also with values obtained for subjects with a history of "long-COVID" but no symptoms of autonomic nervous system involvement. Unfortunately, we could not control the patients who fully recovered in the follow-up in order to assess the normalization of the autonomic function testing. Results of this preliminary study should be confirmed in a large multicentric validation study.

## Conclusion

We show here that, despite late referral to our department, a comprehensive evaluation of sympathetic and parasympathetic functions, and combined neurological and cardiological expertise made it possible to detect a probable involvement of the autonomic nervous system, revealed more often by parasympathetic dysfunction, potentially accounting for the observed disabilities of these patients. The results of this comprehensive evaluation of autonomic function are consistent with the findings of two other studies reporting a similar autonomic imbalance in patients with long-COVID and parasympathetic overtone.

## Data Availability

The datasets used and/or analyzed during the current study are available from the corresponding author on reasonable request.
